# Methodological Considerations for the Use of Acid‐Based Pre‐Treatment Protocols for Carbon and Oxygen Analysis of Tooth Enamel

**DOI:** 10.1002/rcm.10090

**Published:** 2025-08-01

**Authors:** Karolina Varkulevičiūtė, Christine Winter‐Schuh, Cheryl A. Makarewicz

**Affiliations:** ^1^ Institute of Prehistoric and Protohistoric Archaeology University of Kiel Kiel Germany; ^2^ Archaeology Stable Isotope Laboratory University of Kiel Kiel Germany

**Keywords:** carbon isotope, oxygen isotope, chemical pre‐treatment, enamel diagenesis

## Abstract

**Rationale:**

Chemical pre‐treatment is a common methodological step aimed to remove exogenous materials introduced to archaeological tooth enamel in the burial environment through diagenetic processes. However, some of these methods, involving the use of oxidising reagents such as NaClO, H_2_O_2_, as well as weak acids like CH_3_COOH, have been shown to alter the chemical composition and stable isotope values of enamel. Here, we aim to re‐examine the effects of commonly used pre‐treatment protocols on bioapatite *δ* ^13^C and *δ* ^18^O values, and investigate the relationship between diagenetic alteration and measured isotope values, as indicated by pre‐screening using attenuated total reflectance‐Fourier transform infrared (ATR–FTIR) spectroscopy.

**Methods:**

Modern and archaeological samples were subjected to 10 commonly used pre‐treatment protocols that apply NaClO, H_2_O_2_ and/or CH_3_COOH to tooth enamel powders at treatment lengths. Preservation status and diagenetic alteration prior to and after treatment were investigated using ATR–FTIR. *δ* ^13^C and *δ* ^18^O values were measured before and after treatment to determine if different wet chemistry protocols induced isotopic shifts.

**Results:**

The results show that all pre‐treatment protocols imparted shifts in *δ* ^13^C and *δ* ^18^O values of up to ± 1.5‰ in both archaeological and modern samples. Most treated samples display increased crystallinity, likely indicating sample recrystallisation. We suggest that these changes indicate the removal of contamination and diagenetic alteration, and also the dissolution and restructuring of enamel carbonate leading to changes in the in vivo isotope signal.

**Conclusions:**

We discourage the use of H_2_O_2_ and NaClO to remove organic matter from samples as it incurs unwanted changes to the enamel structure and carbon and oxygen isotope ratios. We also recommend the use of only short‐duration acetic acid treatment protocols to avoid recrystallisation caused by prolonged acid exposure and concomitant unwanted change to in vivo isotope values.

## 1 Introduction

Carbon (*δ* ^13^C) and oxygen (*δ* ^18^O) isotope ratios recorded in tooth enamel are routinely measured by ecologists and archaeologists to establish (paleo)environment and vegetation dynamics as well as dietary intake and mobility in humans and animals [1, 2, 3, 4, 5]. For example, questions regarding the introduction of new plant cultivars, such as maize and millet, can be tracked in human and animal teeth carbon isotope values by taking advantage of the strong isotopic distinction between C_3_ and C_4_ plants [6], while oxygen isotope values have been used to investigate human mobility as it directly relates to *δ* ^18^O values of imbibed water of their local residence during the tooth formation process [5]. Oxygen isotope ratios recorded in animal teeth have high potential to generate high‐resolution paleoclimate information at local scales that can be more directly linked to loci of ancient human activity and also provide information about animal mobility.

The accuracy of such dietary, mobility, and palaeoclimate reconstructions, however, is highly dependent on the recovery of in vivo isotope signals from tooth enamel bioapatite. Bioapatite can be altered by several modes of diagenetic alteration. This includes the exogenous introduction of contaminating organic and inorganic compounds [7], enamel structure dissolution [8], and recrystallisation [9, 10, 11] that can take place after teeth enter the archaeological burial environment and, consequently, alter *δ* ^13^C and *δ* ^18^O values. Chemical pre‐treatment of samples, while intended to eliminate or reduce the impact of diagenetic alteration on isotope ratios, can introduce additional changes to the enamel bioapatite and thus change its isotopic composition [12, 13, 14, 15]. Whether caused by diagenetic alteration or pre‐treatment chemistry, shifts in oxygen and carbon isotope ratios away from the original in vivo values can profoundly affect interpretation of results. For example, alterations to in vivo carbon isotope values may result in inaccurate estimation of the contribution of C_4_ plants such as millet to the diet and this, in turn, can lead to differing interpretations of the importance of this cultivar in human subsistence or livestock foddering regimes. Similarly, shifts in oxygen isotope ratios can lead to different conclusions about mobility patterns, seasonality, and local environment.

### 1.1 Tooth Enamel Composition and Diagenesis

Tooth enamel is comprised of a mineral component (> 96%) mostly in the form of hydroxyapatite (Ca_10_(PO_4_)_6_(OH)_2_) and < 1% organic material [16]. Minor elements, such as magnesium (Mg), sodium (Na), chlorine (Cl), and strontium (Sr), are also commonly present, filling vacancies at the calcium (Ca) and hydroxide ion (OH^−^) sites of the hydroxyapatite crystalline lattice [17, 18]. The inorganic phase of tooth enamel consists of flat, hexagonal hydroxyapatite crystallites [19] arranged as micrometre‐sized prisms (or rods) filling the enamel matrix [20]. Within the prism ‘body’, crystallites are densely packed with little interstitial pore space. However, the volume of pore space increases at the ‘tail’ region of the prism and along prism boundaries due to changes in crystal orientation [21, 22]. Porosity within enamel prism structures facilitates the diffusion of water [23] and water‐soluble matter throughout the tooth enamel, including ions and some small organic molecules [24]. Circulation of water in the pore space of bioapatite can cause dissolution of organic and inorganic matter as well as introduce exogenous compounds via ion exchange [25], potentially altering the original crystalline structure of the enamel.

Diagenetic alteration of the bioapatite structure occurs via incorporation of new chemical species, such as exogenous carbonate or minor elements [26, 27, 28], dissolution of structural carbonate [8], or internal restructuring of the bioapatite [29]. Incorporation of non‐biogenic carbonates may occur in carbonate‐rich burial environments where exogenous carbonates precipitate in enamel cracks or as bicarbonate adsorbed to crystal surfaces [26]. Biological molecule degradation initiated post‐mortem increases the number and size of interstitial pores present in the enamel prism structure, which allows fluid from the surrounding environment to penetrate and migrate through the enamel matrix [30]. Consequently, minor elements present in soil burial environments such as magnesium (Mg), iron (Fe), strontium (Sr), and zinc (Zn), diffuse through liquid along prism boundaries and are subsequently incorporated into the extant bioapatite structure [9, 31, 32]. This diffusion of trace elements, such as strontium, into enamel bioapatite has been associated with shifts in the isotopic composition of tooth enamel [33, 34, 35].

However, the impact of minor element leaching on the enamel matrix remains unclear. Enamel crystallinity may be affected and, consequently, carbon and oxygen isotope values. Dissolution and recrystallisation of bioapatite, initiated by changes in burial environment temperature, pH, and microbial action, may shift in vivo stable isotope values [8, 36, 37]. Heating bioapatite removes structurally incorporated water and organic components [38]. Alkaline conditions can promote recrystallisation of bone bioapatite [39]. Meanwhile, microbial activity can break down molecular bonds within the bioapatite, thus accelerating isotope exchange between enamel carbonate and phosphate oxygen and groundwater [40].

The effect of diagenesis on bone and tooth bioapatite can be identified and quantified using attenuated total reflectance–Fourier transform infrared (ATR–FTIR) spectroscopy. Certain characteristic FTIR peaks can indicate the presence of exogenous material. For example, a peak at approximately 710 cm^−1^ is diagnostic of calcite (CaCO_3_), which can occur as a precipitate from soil [41, 42]. FTIR indices variously describe the ordering of bioapatite crystalline structure and carbonate substitutions. The infrared splitting factor (IRSF) describes bioapatite crystallinity and can be useful in identifying heating or recrystallisation. Unaltered enamel typically shows IRSF values between 3.1 and 3.9, while values above 4.0 indicate heating [43, 44] or recrystallisation caused by exposure to groundwater [10, 11, 45, 46, 47]. Other common indices include ratios of A‐type and B‐type carbonate substitutions (type A/type B carbonate to phosphate index, API and BPI) and carbonate to phosphate ratios (C/P). These indices, however, and the diagenetic alteration communicated by these indices may not necessarily mean that concurrent shifts in the isotopic composition of bioapatite from their original in vivo *δ* ^13^C and *δ* ^18^O values took place.

### 1.2 Common Methods to Address Diagenesis

Concern that diagenetic alteration may alter the isotopic composition of the target tissue has precipitated the development and use of various chemical pre‐treatments, each aimed at removing endogenous organic matter, organic contamination, and/or exogenous diagenetic carbonates with the goal of measuring carbon and oxygen isotope ratios that most faithfully reflect original organismal *δ* ^13^C and *δ* ^18^O values (summary in Table 1). Oxidising reagents such as basic sodium hypochlorite (NaClO) are used to remove organic matter in concentrations ranging from 1% to 5% at treatment lengths up to 24 h at room temperature [48, 49, 50]. Reactions of bioapatite with NaClO also appear to cause a dissolution of atmospheric CO_2_, which is converted into carbonic acid followed by bicarbonate and carbonate ions precipitating on the powder sample, thus altering the carbon and oxygen isotope ratios from their original isotope values [12]. A less prevalent, but still popular, oxidising reagent is acidic hydrogen peroxide (H_2_O_2_), applied at 3% to 30% strength with treatment lengths varying from 15 min up to 24 h at different prescribed temperatures [51, 52, 53, 54, 55]. The low pH of H_2_O_2_ causes excessive sample loss [14], problematic for high‐resolution sampling strategies that generate small amounts of material for isotope analysis such as intra‐tooth sequential sampling. The loss of structural carbonate post H_2_O_2_ treatment is also reflected in the decrease of type B carbonate [60, 61], a carbonate replacing a phosphate in the hydroxyapatite structure. Despite these concerns and the negligible organic content in enamel, both reagents are still widely applied to ancient specimens.

**TABLE 1:** A summary of enamel pre‐treatment protocols commonly applied to tooth enamel powder.

Reagent	Purpose	Concentration	Temperature	Treatment length	Concerns	References
NaClO	Removal of organic content	1%–5%	Room temp.	30 min–24 h	Might not fully remove organic contamination, induces absorption of atmospheric CO_2_	[48, 49, 50]
H_2_O_2_	Removal of organic content	3%–30%	Room temp. or heated to 70°C	15 min–24 h	Might not fully remove organic contamination, low pH causes excessive sample loss	[14, 51, 52, 53, 54, 55]
CH_3_COOH	Removal of carbonate contamination	0.1–1 M	Room temp.	10 min–24 h	Removal of structural carbonate, excessive sample loss, recrystallisation	[15, 56, 57, 58, 59]

Exogenous carbonates from tooth enamel powders are usually removed using acetic acid (CH_3_COOH), following foundational experimental work by Lee‐Thorp and van der Merwe [56]. Typically, non‐lattice‐bound and therefore more soluble, exogenous carbonates are easily removed with low‐concentration acids. In recent years, 0.1 M acetic acid and 1 M sodium acetate buffered acetic acid applied at room temperature with treatment length varying from 10 min to 24 h are most commonly used [15, 57, 58, 59], contrasting with the use of 1 M concentration unbuffered acetic acid in the past [62, 63]. Buffered acetic acid is characterised by higher, closer to neutral, pH levels that help conserve sample amount [60]. However, assessing the effectiveness of acetic acid in removing exogenous carbonates is challenging, as these carbonates share a chemical composition and therefore FTIR absorption peaks, with indigenous carbonates. Only the significant presence of calcite, which displays a characteristic peak at 710 cm^−1^ not present in pure enamel hydroxyapatite [41, 42], or extensive recrystallisation of carbonate into brushite [56] can definitively identify diagenetic alteration of carbonate. Furthermore, it appears that structural lattice‐bound carbonates are also lost during pre‐treatment, suggested by sample loss and decreased carbonate concentrations observed in powdered samples from modern specimens that never entered a burial environment [12, 14, 60]. Prolonged acetic acid treatment time can also lead to the recrystallisation of carbonate recrystallisation into brushite [56], although it is likely that some degree of recrystallisation takes place even with shorter treatment durations [13]. Acetic acid treatments have been shown to inconsistently impact *δ* ^13^C and *δ* ^18^O values, with shifts up to approximately ± 1‰ observed [12, 13, 14]. Notably, it is not well understood whether these isotopic shifts are due to the removal of contaminating exogenous carbonates, removal of biogenic carbonates, enamel recrystallisation, or introduction of exogenous matter [12, 14, 15, 59, 60].

As archaeological interpretations of paleoclimate and paleodiet increasingly draw from ever smaller per mil isotopic distinctions between samples to identify fine‐scale changes in environment, mobility, and consumption behavior, obtaining isotope values that most closely reflect their in vivo signal is increasingly important. Here, we present a large scale, systematic re‐examination of the extent to which different pre‐treatment protocols affect the structural integrity, chemical composition, and isotopic composition of tooth enamel bioapatite. We aim to illustrate how different pre‐treatment protocols alter enamel bioapatite and how pre‐treatment led changes may affect measured isotope values, and, thereby influence subsequent palaeodietary, palaeomobility, and palaeoclimatic interpretations.

## 2 Materials

To assess the impact of different pre‐treatment protocols, a total of 30 archaeological and 10 modern sheep/goat ( *Ovis aries* / *Capra hircus* ) and cattle ( *Bos taurus* ) mandibular molar teeth (M/2 and M/3) were sampled. We have chosen to investigate sheep/goat and cattle as they are two of the most commonly found species in the archaeological record in Eurasia as well as most commonly investigated taxa in enamel stable isotope studies. Archaeological sites of different ages, geographic, and climatic contexts were chosen to explore how these factors might impact enamel bioapatite response to pre‐treatment. Based on visual inspection, selected specimens displayed varying states of preservation. In addition to archaeological specimens, modern faunal material was chosen as a reference against which diagenetically altered material could be evaluated. Archaeological material was obtained from three sites: Tell Handaquq South (Jordan), Wadi Faynan 16 (WF16, Jordan), and Oldenburg LA 77 (Germany). Modern samples were selected from two sites: Olfen (Germany) and Baga Gazaryn Chuluu (Mongolia).

Tell Handaquq South (THS; *n* = 10; five *Ovis/Capra*, five *Bos*) is a large Early Bronze Age settlement site (3600–2000 cal bc) located on a slope abutting the southern bank of the Wadi Zarqa at the base of the Jordanian highland escarpment [64]. Regional paleoclimate studies suggest the site experienced semi‐arid conditions with estimated precipitation levels of approximately 350 mm per annum [65, 66]. Calcic soils characterise the area around the settlement [67], and while no sediment analyses have been carried out at the site, most of the deposits are described as silty in the excavation report [68]. The majority of the bones and teeth recovered from Tell Handaquq South were encrusted with calcareous concretions of up to a few millimetres in thickness and required mechanical removal prior to the tooth sampling procedure (Figure S1A).

Oldenburg LA 77 (OLD; *n* = 10; four *Ovis/Capra*, six *Bos*) is a large Middle Neolithic Funnel Beaker settlement site (ca. 3300–2800 bc) located on the Baltic coast on the Wagrien peninsula in Schleswig‐Holstein, northern Germany [69]. The site is situated on the waterfront wetland area within the Oldenburger Graben glacial valley and, during occupation two former fjord‐like estuaries (Oldenburg and the Gruber Förde) formed lagoons with varying water table and salinity levels [70, 71]. The climate in the region during the Neolithic was characterised by cool temperatures and wet conditions [72, 73]. The peripheral areas of the site support peat formations, calcareous, detritus‐rich mud with sand inclusions [71]. ATR–FTIR analysis of the sediment indicates a high silica content (likely quartz), gypsum, and only very low quantities of carbonates (Figure S4A). Oldenburg LA 77 specimens sampled here display brown and, sometimes, almost black staining (Figure S1B). Such staining on bone and teeth, common for specimens recovered from waterlogged contexts, is likely due to contact with dark organic matter, iron, and manganese compounds found in soil [74]; high concentrations of iron and manganese oxides are often found in such teeth [75, 76, 77].

Wadi Faynan 16 (WF16; *n* = 10; five *Capra* sp., five *Bos primigenius* ) is a Pre‐Pottery Neolithic A settlement (10 070–8160 cal bc) [78, 79] located at the head of the Wadi Faynan, a seasonally flooded water course between the rocky Sharah mountains rising up to the Jordanian plateau (ca. 1300 m a.s.l.) and the arid flats of Wadi Araba (ca. 0 to 200 m a.s.l.). Hydrological simulations suggest that at the beginning of the Holocene (ca. 12 000 bc), Wadi Faynan experienced similar, albeit slightly wetter, conditions than the present‐day climate, characterised by rapid flood responses to rainfall events in the winter season [80]. Sedimentology work shows that sediments recovered from the archaeological deposits were sandy, alkaline, and primarily minerogenic in nature, characterised by varying proportions of quartz, calcite, gypsum, and a low organic content [81]. This is further confirmed by FTIR analysis of sediment, recovered with the bone specimens, that demonstrate a high silica and carbonate content with the presence of quartz and gypsum (Figure S4B). The animal teeth recovered from WF16 present as highly fragmented with thin and brittle enamel (Figure S1C).

Modern sheep teeth (*n* = 5) were chosen from an ethnographic site located in the Mongolian Gobi steppe desert at Baga Gazaryn Chuluu (BGC; Dundgovi *aimag*). Animals died in 2000–2001 during a *dzud* event, an extremely cold winter accompanied by snow or ice that removes or covers graze so that livestock starve. Summers are hot, with most precipitation falling during the summer months, and winters severely cold [82]. Specimens were exposed until collection in 2003 and bore signs of weathering. Modern cattle teeth (*n* = 5) were sourced from Olfen (OLF) located in North Rhine‐Westphalia, western Germany, from animals slaughtered in 2018–2019. The cattle were largely unmanaged and kept in a grassy pasture field year‐round with some hay supplementation during the winter months. The region is characterised by a mild temperate climate, with an average annual temperature of 10°C (≈ 18°C in July and ≈ 5°C in January) and mean annual precipitation of 850 mm.

## 3 Methods

Prior to sampling, the tooth enamel surface was cleaned from soil and concretions using a dental burr, followed by ultrasonication in ultrapure water and then dried. This was particularly important for teeth from THS which were covered in thick calcareous concretions; removed concretions were set aside for later ATR‐FTIR analysis. The length of the tooth lobe was cut, and dentine was removed using a diamond‐tipped Dremel drill. Dentine is characterised by a different colour and hardness compared with enamel, and full removal of the dentine was ensured through visual inspection under good lighting conditions. Clean enamel was ground into a homogenous powder using a Retsch MM40 mixer mill. Prior to experimental treatment, each enamel sample was screened using ATR–FTIR spectroscopy on a Bruker Alpha II ATR–FTIR at the Archaeology Stable Isotope Laboratory, Institute of Prehistoric and Protohistoric Archaeology, University of Kiel. Around 1 mg of sample (enough to cover the crystal) was measured twice, and the spectra from 450 to 4000 cm^−1^ were collected for 100 scans at a resolution of 4 cm^−1^. Baseline correction and peak picking were carried out using the Bruker OPUS 8.7 software. The following indices were calculated to characterise the composition and structure of the enamel and assess any diagenetic alteration present in the samples: infrared splitting factor (IRSF), ratios of A‐type (substituting the OH group), and B‐type (substituting the phosphate) carbonate to phosphate peaks (API and BPI), carbonate to phosphate ratio (C/P), and carbonate to carbonate ratio (C/C) (Table 2). Guidelines for accepted ranges for ‘well‐preserved’ enamel were taken from France et al. [83], which combined the observations of modern and historic samples that passed visual analyses, weathering indices, and organic collagen preservation indicators. While collagen preservation does not equate to the preservation of the in vivo carbonate‐derived isotope values, it is still a useful control measure in the absence of other carbonate preservation indicators [83].

**TABLE 2:** ATR‐FTIR indices used in this study, along with corresponding peak height ratios, significance, and the suggested ratios for “well‐preserved” enamel (based on France et al. [83]).

Name	Peak height ratios (cm^−1^)	Significance	“Well‐preserved” range
IRSF	(565 + 605)/590	Indicator of crystallinity	3.1–3.9 (untreated) 3.1–4.0 (chemically treated)
C/C	1455/1415	Compares two carbonate bands	0.9–1.1
C/P	1415/1035	Carbonate to phosphate ratio	0.08–0.2
API	1540/605	Amount of A type carbonate substitutions	0.04–0.2
BPI	1415/605	Amount of B type carbonate substitutions	0.1–0.65

In this study, 11 different treatment protocols were tested, including an untreated control group. Protocols included organic removal pre‐treatment with H_2_O_2_ and NaClO as well as secondary carbonate removal pre‐treatment with different concentrations of acetic acid (Table 3). For each treatment, 20 mg of powder was subsampled and treated with around 1.5 mL of solution, and 15 mg of enamel powder was subsampled for the untreated group.

**TABLE 3:** Pre‐treatment protocols investigated in this study.

Label	Reagent	Concentration	Time	T (°C)	Justification	Previous applications
	No treatment					
15 min H_2_O_2_	H_2_O_2_	3%	15 min	Room temp.	Oxidising agent used for organic contamination removal (e.g., humic acids) in bone mineral, subsequently used for enamel preparation	[51, 52, 53, 84]
45 min NaClO	NaClO	3%	45 min	Room temp.	Oxidising agent used for organic contamination removal (e.g., humic acids) in bone mineral, subsequently used for enamel preparation	[49, 85, 86, 87]
16 h NaClO			16 h			
15 min unbuff	CH_3_COOH	0.1 M	15 min	Room temp.	Exogenous carbonates are more soluble than apatite and can be removed by leaching in dilute acid, such as CH_3_COOH	[49, 56, 85, 86]
4 h unbuff			4 h			
8 h unbuff			8 h			
15 min buff	(CH_3_COOH)_2_Ca	1 M buffered	15 min	Room temp.	Buffering the acid makes it closer to neutral pH and less destructive to the sample	[12, 60, 86]
4 h buff			4 h			
8 h buff			8 h			
NaClO + Acetic	NaClO + CH_3_COOH	3% + 1 M buffered	45 min + 15 min	Room temp.	Combining the organic removal step and carbonate exogenous carbonate removal protocols gets rid of all unwanted matter	[49, 85, 86, 87]

Three organic removal treatment protocols were chosen for this study based on the most commonly cited protocols in the literature. Group 1 received a 3% H_2_O_2_ pre‐treatment applied at room temperature for 15 min. Groups 2 and 3 received a 3% NaClO pre‐treatment at room temperature for 45 min and 16 h, respectively. Treatment lengths were selected to capture the large range in duration of treatment, from 30 min to several days, described in the recent literature [86, 88, 89].

Secondary carbonate removal was tested with 0.1 M unbuffered acetic acid (CH_3_COOH) and 1 M calcium acetate buffered acetic acid ((CH_3_COOH)_2_Ca), two commonly used reagents used for this purpose [15, 56, 57, 58, 59]. These two types of acetic acids were also chosen to try and replicate the differences in mass loss resulting from pre‐treatment previously investigated by Snoeck and Pellegrini [60]. Three treatment durations were selected for both types of acetic acid protocols: 15 min, 4 h and 8 h.

The last treatment protocol used here is a combined protocol of 45 min 3% NaClO treatment followed by a 15‐min 1 M buffered acetic acid treatment (i.e., [49, 86, 90, 91, 92]). The 15‐min buffered acetic acid treatment has been chosen for the second step as previous experimental work has shown that buffered acetic acid appears to reduce sample loss compared to the unbuffered acetic acid treatments [12, 13, 60].

After pre‐treatment, all of the sample powders were rinsed with ultra‐pure water five times and freeze‐dried prior to isotope ratio mass spectrometry. Mass loss caused by pre‐treatment was determined by weighing each sample prior to and after the treatment. Supernatant remaining after the treatment and rinsing was dried and analysed using a Bruker Alpha II ATR–FTIR at the Archaeology Stable Isotope Laboratory, Institute of Prehistoric and Protohistoric Archaeology, University of Kiel.

Stable oxygen and carbon isotope ratios of powdered enamel carbonates were analysed using a MAT 253 dual‐inlet isotope ratio mass spectrometer coupled to a Kiel IV carbonate device at the Leibniz‐Laboratory for Radiometric Dating and Stable Isotope Research, University of Kiel. Samples were reacted by individual acid addition (99% H_3_PO_4_ at 75°C), and the evolved carbon dioxide was analysed eight times for each sample. Isotope ratios are reported in permil (‰) using the delta (*δ*) notation and calibrated relative to the Vienna PeeDee Belemnite (VPDB) scale. The *δ* notation refers to a measure of deviation in ratio between a heavy and a light stable isotope (in this case ^18^O/^16^O and ^13^C/^12^C), and the result expresses the relative difference between a sample and a standard in parts per thousand (‰).

δO18=O18O16sampleO18O16standard−1×1000

δC13=C13C12sampleC13C12standard−1×1000

Analytical precision was ±0.08‰ for both *δ* ^18^O and *δ* ^13^C, based on the performance of international standards (NBS19: *δ* ^13^C +1.95‰, *δ* ^18^O −2.20‰; IAEA‐603: *δ* ^13^C +2.46‰, *δ* ^18^O −2.37‰) and internal laboratory pure carbonate standards (Hela *δ* ^13^C +0.91‰, *δ* ^18^O +2.48‰; SHK: *δ* ^13^C +1.74‰, *δ* ^18^O −4.85‰; HB1: *δ* ^13^C −12.10‰, *δ* ^18^O −18.10‰). Additional laboratory internal enamel bioapatite standards (ER1: *δ* ^13^C −12.18‰, *δ* ^18^O −7.78‰; ES1: *δ* ^13^C −13.76‰, *δ* ^18^O −6.41‰) yielded a standard deviation of 0.07‰ for *δ* ^13^C and 0.10‰ for *δ* ^18^O (ER1), and 0.07‰ for both *δ* ^13^C and *δ* ^18^O (ES1). Sample duplicates were run on average every 10 samples (*n* = 84), showing an average deviation of ±0.05‰ for *δ* ^13^C and ±0.16‰ for *δ* ^18^O.

Concretions removed from THS teeth were ground into a homogenous powder. A subsample of the powder was analysed using ATR–FTIR spectroscopy and isotope ratio mass spectrometry to determine the chemical composition and well as *δ* ^13^C and *δ* ^18^O values of the concretions. Sediments adhering to the Oldenburg LA 77 and WF16 teeth or sediments remaining in the sample bags were also selected and analysed using ATR–FTIR spectroscopy to determine the chemical composition of the burial environment.

## 4 Results

### 4.1 Mass Loss

**FIGURE 1:**
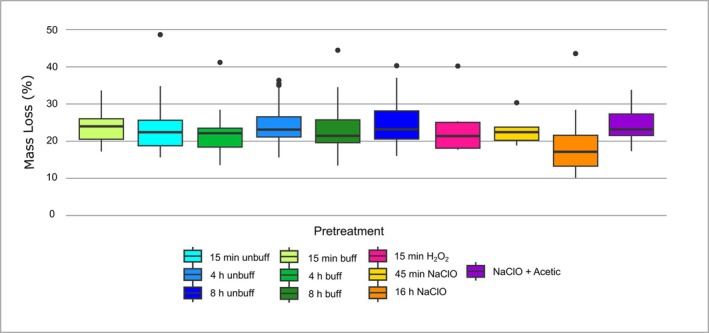
Sample weight loss after treatment according to pre‐treatment protocol and treatment duration.

Sample loss due to treatment protocol type is expressed here as a percentage loss of dried sample calculated relative to the sample weight prior to treatment (see Figure 1). All treatment protocols caused a notable mass loss in both archaeological and modern samples ranging on average from 16.3% (3% NaClO 45 min) to 24.6% (4 h unbuffered CH_3_COOH). There was no correlation observed between the treatment length and sample loss in both buffered and unbuffered acetic acid treatments (*r* = −0.13 and *r* = 0.084, respectively). A nonparametric Kruskal–Wallis test followed by a post hoc Dunn's test (see Table S5) showed that only samples treated with 3% NaClO for 45 min were statistically different from the other treatment groups with an average mass loss of 18.3% compared with a mass loss of 22–24% observed in other groups. Outliers are present in all of the treatment groups; the higher sample loss in the outliers are likely due to static in the sample tubes during freeze drying, storage, and weighing.

### 4.2 Sample Preservation Parameters Prior to Treatment

**FIGURE 2:**
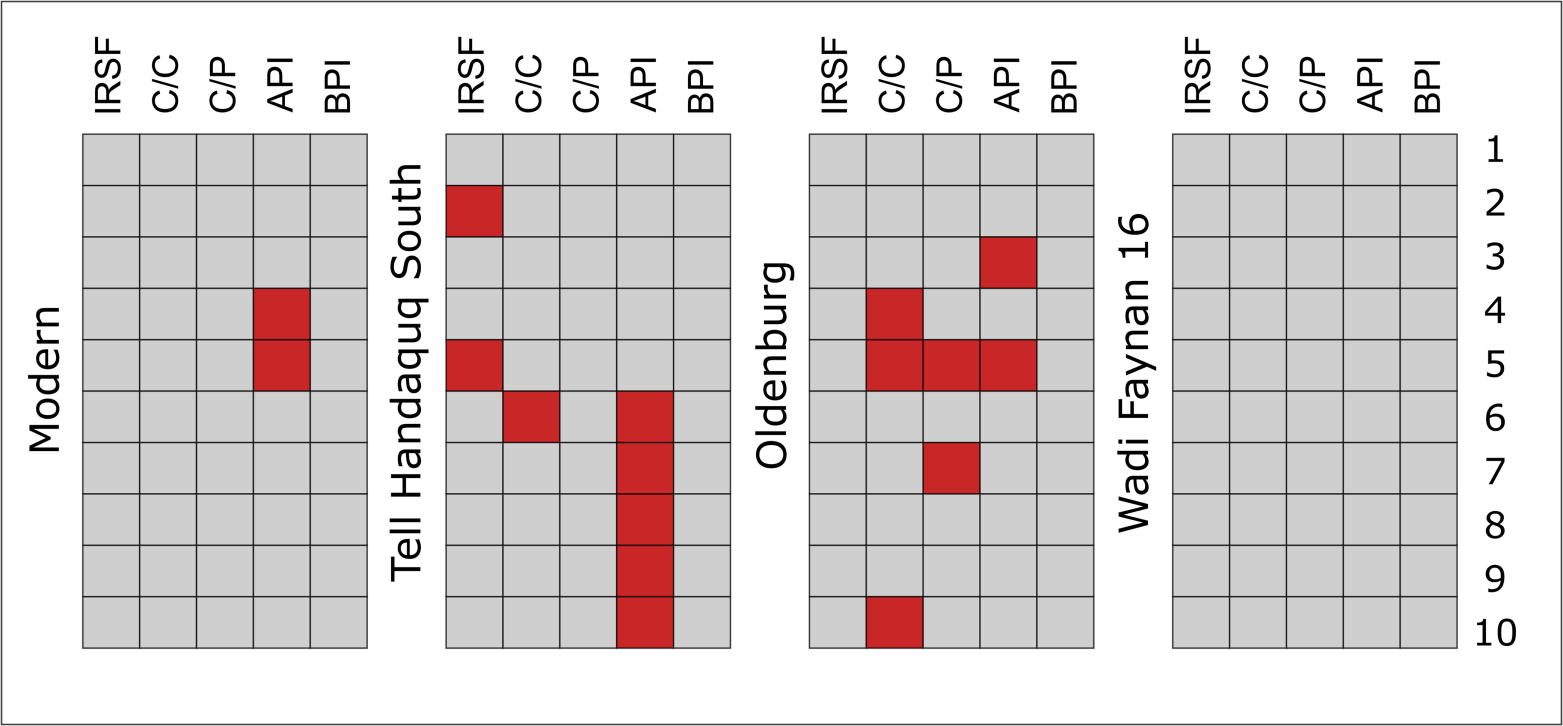
Summary of FTIR parameter pre‐screening results. Each box represents a powdered enamel sample from an individual tooth specimen (numbers 1 through 10) according to site: Modern (BGC and Olfen), Tell Handaquq South, Oldenburg LA 77, and Wadi Faynan 16. Grey boxes indicate that the powdered sample yielded peak height ratios within the accepted range for well‐preserved enamel; red boxes indicate the sample yielded peak height ratios outside the accepted range for specific parameters presented in Table 2.

Sample preservation was assessed through the calculation of five commonly used FTIR indices: IRSF, C/C, C/P, API, and BPI, and the ranges for ‘well‐preserved’ enamel published in France et al. [83] (see Table 2). The majority of the samples were within the accepted range for all indices (Figure 2; Table S2). The API index, which represents ratios of A‐type carbonate substitutions to phosphate peaks, was most frequently expressed outside of accepted range, visible in nine out of 40 samples. This included two modern specimens from BGC which was unexpected, as modern samples should not be diagenetically altered. The C/C index, the ratio between type A and type B carbonates, yielded values above the accepted range of 1.1 for four out of 40 samples. Values outside of the accepted range for C/P, the ratio between carbonate and phosphate peaks and IRSF, crystallinity factor, were observed in two out of 40 samples. For samples THS2 and OLD5, IRSF values were 4.0 and 4.3, respectively, considerably above the accepted 3.1–3.9 range for untreated enamel and much higher compared to all other specimens, which on average yielded values of 3.5. The higher IRSF values indicate a more ordered crystal structure within enamel, which could be a result of carbonate alteration, ionic exchange during burial, microbial activity, or recrystallisation caused by heating or natural recrystallisation during burial/groundwater exposure [10, 11, 43, 44, 45, 46, 47].

Modern samples from OLF and BGC yielded acceptable preservation parameters. Two samples from BGC failed the API index but this could be related to visible weathering in the specimens or the occasionally indistinct peak at 1540 cm^−1^ peak used in the calculations of this parameter. All of the samples from WF16 yielded preservation parameters within the accepted range for all five indices, suggesting good preservation of the enamel despite outstandingly poor dentine collagen preservation. Samples from THS and Oldenburg LA 77 yielded the highest proportion of samples, seven and five, respectively, outside of accepted ranges. The 1415 cm^−1^ peak used in the calculation of C/C, C/P and BPI indices, all of which yielded values outside accepted ranges at THS and Oldenburg LA 77, is a prominent peak also observed in pure CaCO_3_, MgCO_3_, and other carbonates commonly found in soil [83]. The 1455 cm^−1^ peak, used in the calculations of C/C index, often overlaps with secondary (diagenetic) carbonates [83]. This coincides well with the visual observations of samples from these sites. Calcareous concretions covering the THS specimens may have promoted precipitation of exogenous carbonate into the enamel structure. Brown discolouration of the Oldenburg LA 77 teeth, commonly observed in specimens from waterlogged contexts, likely results from the absorption of manganese and iron oxides from the surrounding soil [74, 75, 76, 77], and may also signal post‐depositional diagenesis.

### 4.3 Effects of Pre‐Treatment on the *δ* ^13^C and *δ* ^18^O Values of Enamel

**FIGURE 3:**
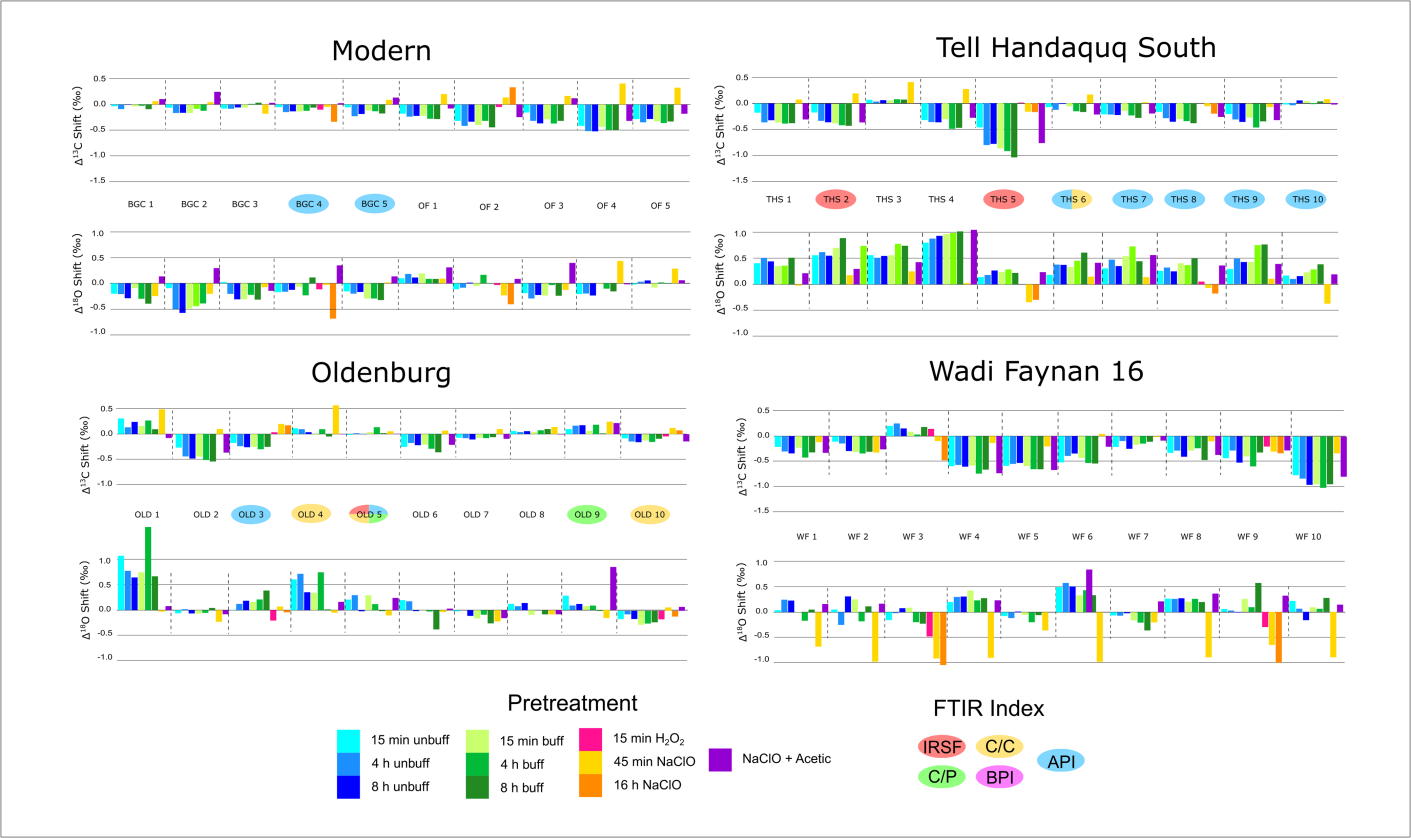
Shifts in Δ^13^C and Δ^18^O values after application of different experimental pre‐treatment protocols. An oval laid over the sample name indicates the FTIR parameters outside the accepted range for “well‐preserved” enamel prior to treatment for that sample.

To assess the isotopic shifts induced by different treatment protocols, isotope values measured from pre‐treated samples were subtracted from those of untreated samples (Δ_isotope_ = *δ*_treated_—*δ*_untreated_). Positive values indicate an increase in *δ* ^13^C or *δ* ^18^O values, while negative values correspond to a decrease in *δ* ^13^C or *δ* ^18^O values (Figure 3; Table S1).

#### 4.3.1 Effects of Organic Removal Pre‐Treatments on Isotope Composition

For H_2_O_2_ treated samples, virtually no carbon isotopic shift was observed, with Δ^13^C values ranging from −0.2‰ to +0.1‰. NaClO, on the other hand, had a stronger and more variable effect on carbon isotope values. Oldenburg LA 77 Δ^13^C values were positive post‐treatment (range ∆^13^C = +0.1‰ to +0.6‰), while WF16 Δ^13^C values were mostly negative (range ∆^13^C = −0.4 to 0.0‰). For THS and modern samples, isotopic shifts in both directions were observed (range Δ^13^C = −0.2‰ to +0.4‰ and −0.3‰ to +0.4‰, respectively). Sample powders subjected to both a 45 min and a 16 h NaClO pre‐treatment exhibited slightly larger shifts in Δ^13^C values for the 16 h treatment compared to the 45 min treatment, with differences of up to 3‰. All organic removal pre‐treatment protocols imparted carbon isotope shifts of up to 0.6‰ and there were no predictable patterns in the range or directionality of change in Δ^13^C values shared between samples from specimens from different sites.

Δ^18^O values were mostly negative for samples treated with H_2_O_2_ (range Δ^18^O= −0.4‰ to 0.1‰) and 16 h NaClO (−0.9‰ to 0.0‰) across all sites. The 45 min NaClO treatment resulted in both positive and negative Δ^18^O values ranging from −0.4‰ to +0.2‰ at THS, −0.2‰ to +0.1‰ at Oldenburg LA 77, −0.9‰ to −0.2‰ at WF16, and −0.2‰ to +0.4‰ for modern samples. When the shorter 45 min and longer 16 h NaClO treatment was applied to the same enamel powder, ∆^18^O values were up to 0.6‰ larger for the 16 h treatment compared to the 45 min treatment. For samples treated with NaClO, the shift in Δ^18^O values was minimal in most modern, THS, and Oldenburg LA 77 samples. In sharp contrast, samples from WF16 were strongly affected by the oxidising organic removal pre‐treatment protocol, with a shift of −0.8‰ or more visible in six out of 10 samples.

#### 4.3.2 Effects of Secondary Carbonate Removal Pre‐Treatments on Isotope Composition

The change in carbon isotope ratios observed post‐acetic acid treatment for specimens from all sites resulted in negative Δ^13^C values, with the exception of Oldenburg LA 77 where the direction of isotopic change varied between individual tooth specimens. Shifts in Δ^13^C values between 0.1 M unbuffered and 1 M buffered acetic acid solutions was minimal, in general below ±0.3‰. There was no observable correlation between treatment length and the amplitude of isotopic change in both the buffered and unbuffered acetic acid treated groups. The shifts in Δ^13^C values observed in the Oldenburg LA 77 and BGC modern specimens were smaller and generally negligible compared with the other sites. For specimens from THS, WF16, and Olfen, shifts in Δ^13^C values generally ranged from −0.2‰ to −0.5‰, with a few isotopic outliers visible across the specimens.

The directionality of oxygen isotopic change visible in samples varied by site, with a positive shift visible in Δ^18^O values (+0.1‰ to +1.0‰) at THS, a negative shift (−0.6‰ to +0.2‰) observed in modern specimens, and specimens from both Oldenburg LA 77 (−0.4‰ to +1.6‰) and WF16 (−0.3‰ to +0.5‰) exhibiting both negative and positive shifts in ∆^18^O values. While there was a wide range in the amplitude of oxygen isotopic change visible for samples treated with buffered and unbuffered acetic acid (ranging from ±0.0‰ to ±1‰), the majority of the samples, regardless of treatment length or type of acetic acid used, exhibited minimal isotopic differences ± 0.5‰ or lower. Only samples from THS exhibited more pronounced oxygen isotopic differences between buffered and unbuffered acetic acid treatments.

#### 4.3.3 Effect of Combined Pre‐Treatment on Isotope Composition

The direction and magnitude of carbon and oxygen isotopic change in samples treated with combined NaClO and acetic acid protocols are similar to those observed for acetic acid‐only treatments. However, combined treatment shifts Δ^13^C and Δ^18^O values in a positive direction while the NaClO‐only treatment shifts Δ^13^C and Δ^18^O values in a negative direction. Isotopic shifts in the combined treatment group vary widely across individual specimens (range = −0.8‰ to +1.0‰), and, no site‐specific patterns are visible.

#### 4.3.4 Effects of Pre‐Treatment on Enamel ATR–FTIR Spectra

**FIGURE 4:**
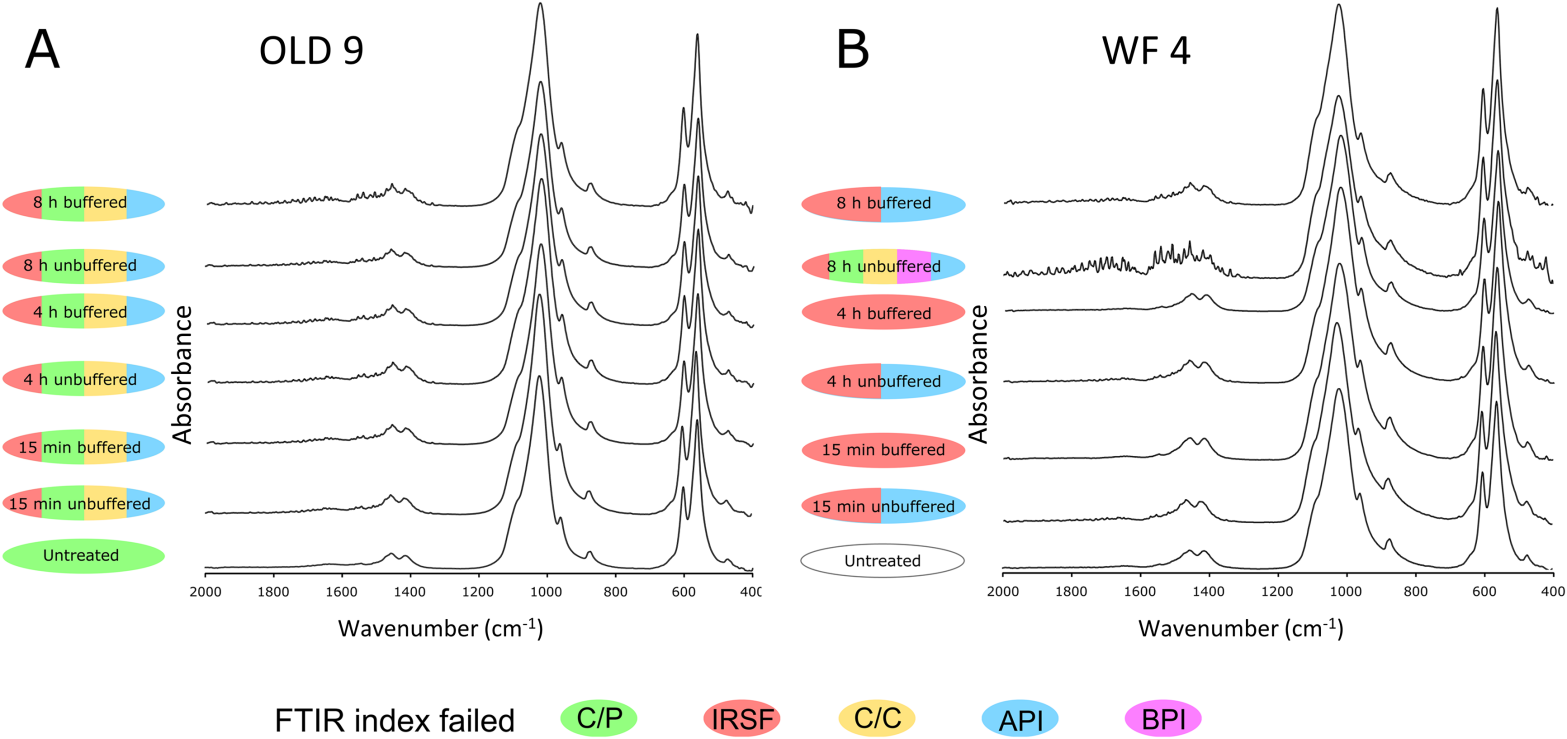
Examples of ATR‐FTIR spectra exhibiting various modes of diagenetic alteration for powdered enamel bioapatite samples subjected to different acetic acid treatments from (a) Neolithic Oldenburg LA 77 (OLD 9) and (b) PPNA WF16 (WF 4). Colours indicate failed FTIR preservation indices: C/P, IRSF, C/C, API, BPI.

The effect of different treatments on ATR–FTIR spectra is visible in a few of the samples. Samples from Oldenburg LA 77 and WF16 yielded distinctly distorted spectra after pre‐treatment (Figure 4). Most samples also failed one or more FTIR parameters post‐treatment, even those that initially yielded acceptable preservation parameters prior to pre‐treatment (Table S3). During post‐treatment, the majority of the analysed samples IRSF, C/C, and BPI parameters fell outside of the accepted ranges after the acetic acid (buffered and unbuffered, 15 min, 4 h, 8 h), NaClO, and combined treatments. This includes both modern and archaeological samples, suggesting that the changes are caused by alterations of the indigenous enamel structures and not just removal of contamination. In almost all samples (over 90%), IRSF values increased up to 0.7 after treatment. Samples that failed FTIR parameters prior to treatment generally showed no significant improvement or decline following pre‐treatment.

## 5 Discussion

### 5.1 Pre‐Treatments for Removing Organic Matter From Bioapatite

Organic removal protocols using H_2_O_2_ or NaClO are regularly used to prepare tooth enamel samples for stable isotope analysis. However, our results, together with previously published studies, demonstrate that the use of oxidising reagents to remove organic contamination from enamel samples is not recommended. Tooth enamel contains a very small amount of organic content (< 1%) compared to 20–25% in dentine and bone [18]. Considering that the organic content in teeth and bone is comprised largely of collagenous proteins that degrade in soils and sediments over time [93], the organic content present in archaeological enamel samples is negligible. Furthermore, previous work has demonstrated that only samples with a high organic content, where the total inorganic carbon to total organic carbon ratio exceeds 0.3, are likely to exhibit meaningful shifts in carbonate oxygen and carbon isotope ratios [94].

The use of an oxidising reagent step for organic removal during enamel sample preparation, however, may alter the chemical properties of the enamel. Here, samples treated with NaClO exhibited significantly less weight loss compared to those subjected to other treatment protocols, with an average mass loss of 18.3% compared to 22–23% documented for other treatments. The lower sample loss could be attributed to adsorption of exogenous carbon from the atmosphere [14, 15, 95]. The high pH of the NaClO solution promotes the dissolution of atmospheric CO_2_, which is subsequently converted into carbonic acid and then bicarbonate and carbonate ions, which can precipitate as secondary calcium carbonate (CaCO_3_) onto the powdered sample [12]. This precipitation of secondary carbonates is further corroborated by previous studies reporting higher carbonate content (%CO_3_) in sodium hypochlorite‐treated samples compared to untreated controls [15, 60, 95].

### 5.2 Pre‐Treatments to Remove Secondary and Exogenous Carbonates From Bioapatite

The results of this study demonstrate that the rate of sample dissolution is similar between 0.1 M acetic acid and 1 M calcium acetate buffered acetic acid treatments. Unbuffered acetic acid has a low pH value (∼ 3) which may promote sample loss, particularly problematic for sequentially sampled teeth where sample yields are already low. Snoeck and Pellegrini [60] found that unbuffered acetic acid imparts a drastically larger sample weight loss, with an average enamel sample loss of 65% compared with a 30% sample loss associated with buffered acetic solution after only a 30 min treatment length. In contrast, our results show that there are no significant differences in the rate of dissolution in tooth enamel samples between buffered and unbuffered acetic acid solutions regardless of treatment length, with an average sample loss of 23% for both treatments. Given the absence of significant differences in sample weight loss across treatment durations, it is plausible that most dissolution occurs within the first 15 min of the reaction. This observation is consistent with findings by Garvie‐Lok et al. [58] and Skippington et al. [13], who reported that enamel samples exhibit the highest reactivity during the first 15 min of treatment, with dissolution equilibrium reached after 4 h of treatment.

The absence of a correlation between treatment duration and the magnitude of carbon and oxygen isotope change in samples treated with unbuffered acetic acid (r = 0.08), along with only a weak negative correlation in buffered acetic acid‐treated samples (r = −0.13), suggests that more than just diagenetic matter is being removed from the samples.  The smaller Δ^13^C and Δ^18^O values after 4 h or 8 h treatment durations compared to the 15 min treatment may indicate an initial dissolution of exogenous (diagenetic) carbonate followed by slower modification of structural bioapatite through dissolution or recrystallisation [13]. As diagenetic carbonates tend to be more soluble, non‐lattice bound ions would dissolve first causing the initial change in bioapatite isotope composition [15]. Any subsequent modification of bioapatite, including dissolution of structural carbonate or recrystallisation, can affect carbon and oxygen isotope values, as reflected in Δ^13^C and Δ^18^O values observed after 4 h and 8 h of treatment [15, 56, 57]. It is important to note, however, that in some samples these shifts are negligible or very small. Given that the 1σ analytical uncertainty, calculated as the standard deviation of repeated measurements of both internal enamel standards and sample duplicates, is 0.1‰ for *δ* ^13^C and 0.2‰ for *δ* ^18^O, these observed small isotopic shifts are not considered significant.

Experimental data measured from modern tooth enamel samples demonstrate that *δ* ^13^C and *δ* ^18^O values of samples change after treatment with acetic acid solution [12, 13, 15, 56]. As modern tooth samples have not been subjected to potential contamination from a burial environment and have not undergone diagenesis, the isotopic changes observed in other studies and here cannot be assigned to the removal of exogenous carbonates. This demonstrates complex processes take place during pre‐treatment, including dissolution of exogenous carbonates and simultaneous reactions with structural carbonate (dissolution and/or recrystallisation) that affects the fidelity of the original in vivo isotope signal.

Changes in FTIR IRSF, C/C, and API indices following acetic acid treatment indicate alterations to the bioapatite structure caused by the treatment itself. An increase in IRSF, an indicator of crystal structural order, is expected with the removal of secondary, diagenetic, and non‐lattice bound carbonate ions [14, 29, 83]. However, it is notable and concerning that several samples exhibiting pre‐treatment IRSF values within the accepted range (3.1–3.9) exhibited elevated IRSF values post‐treatment, ranging from 4.1 to 4.6. Although FTIR spectra of untreated and chemically treated bioapatite are not directly comparable due to differing preparation methods [83, 96], the magnitude of change observed in IRSF values suggests that chemical alteration beyond the removal of contaminants is occurring during treatment. Specifically, the increase in IRSF values is consistent with recrystallisation processes, which produce a more ordered crystalline structure. While our study did not observe brushite recrystallisation (see Lee‐Thorp & van der Merwe [56]), the high IRSF (> 4.0) values nonetheless indicate that some degree of recrystallisation is likely taking place during acetic acid exposure [97]. This warrants caution in interpreting isotopic data from treated samples, particularly when FTIR indices shift beyond established thresholds. When the supernatant from the acetic acid treatment is dried and analysed using ATR‐FTIR, it becomes evident that structural components of the powdered enamel sample are being dissolved. The FTIR spectra of the supernatant closely resemble that of enamel bioapatite, exhibiting characteristic phosphate peaks at 1015, 565, and 605 cm^−1^ as well as carbonate peaks at 1415, 1450, and 1550 cm^−1^ (Figure S2). Notably, supernatant peak intensities, particularly the 1550 cm^−1^ peak in carbonate region of the spectra, differ significantly from those of raw enamel powder. This difference likely reflects the differential dissolution of type‐A, OH‐ substituting carbonate associated with the 1450 and 1550 cm^−1^ peaks versus type‐B, phosphate‐substituting carbonate associated with the1415 cm^−1^ peak. Type A carbonate ions may dissolve more readily due to their larger size and greater structural disorder relative to the OH‐ ions they replace [83]. Additionally, Amide I and Amide II bands, indicative of organic material, appear in the 1400–1700 cm^−1^ range. While some spectral variation may be attributable to the removal of residual organics, the overall organic content in enamel bioapatite is minimal and does not sufficiently account for the pronounced 1550 cm^−1^ peak. These findings suggest that structural components, including carbonate substitutions, are being partially dissolved during acetic acid treatment.

### 5.3 Combined Organic and Secondary Mineral Pre‐Treatment

When acetic acid is applied as a secondary treatment step following NaClO pre‐treatment, the directionality in carbon isotopic change is the opposite of that observed for the NaClO‐only pre‐treatment. The combined treatment also results in the same weight loss comparable to that observed for the acetic acid‐only treatment, whereas NaClO treatment alone yields a lower average weight loss of approximately 16%. The application of sodium hypochlorite to enamel samples induces dissolution of atmospheric CO_2_, which subsequently precipitates as carbonate ions in the solution, it is therefore likely that the subsequent acetic acid treatment removes these precipitated carbonates [12, 60, 98]. Since these precipitated carbonates are not structurally bound to the enamel bioapatite, they would be dissolved along with any diagenetic carbonates. However, complete removal of all precipitated carbonates during this step cannot be guaranteed. Given the negligible organic content in tooth enamel and the potential for secondary carbonate precipitation, the application of NaClO, either alone or in combination with acetic acid, should be avoided in enamel sample preparation.

### 5.4 Does Burial Environment Affect the Preservation of the Isotopic Signature?

FTIR indices indicate that, regardless of burial environment, archaeological tooth enamel samples in this study have been affected by diagenesis or other transformation processes. At THS, the presence of calcic [67] soils likely contributed to the precipitation of calcareous concretions on tooth enamel surfaces, which FTIR analysis identified as carbonated apatite (Figure S3). FTIR screening of the THS enamel powders also showed some diagenetic alteration in the enamel structure, possibly related to these concretions. The average *δ* ^13^C and *δ* ^18^O values of the concretions, −8.7‰ and −2.0‰, respectively (Table S4), closely resemble those of the corresponding untreated enamel sample, suggesting possible contamination by carbonated apatite. Following acetic acid treatment, enamel *δ* ^13^C values were depleted in ^13^C and enriched in ^18^O, which may indicate contamination originating from the concretions was removed. However, since *δ* ^13^C and *δ* ^18^O values of untreated enamel and adhering concretions were so similar, it is also possible that the isotope value change is due to the removal of structural carbonate during acetic acid treatment rather than removal of contamination. Supporting this interpretation, samples from WF16 recovered from a similarly carbonate‐rich environment but lacking visible concretions or FTIR signatures indicating diagenetic alteration, also exhibited negative *δ* ^13^C shifts of a similar magnitude and positive δ^18^O shifts of smaller magnitude as the THS samples. This pattern suggests that isotopic changes associated with chemical pre‐treatment may primarily result from partial dissolution and/or recrystallisation processes induced by the pre‐treatment itself, rather than removal of diagenetic carbonates.

The Oldenburg LA 77 samples appear to have undergone a distinctly different diagenetic pathway compared to those from THS and WF16, likely due to fluctuations in the local water table and the relatively low carbonate content of the burial environment sediments. The brown to black staining present on the teeth is consistent with contamination by iron and manganese oxides which is commonly found in waterlogged dental and osseous tissues [74, 75, 76, 77]. Water movement through pore space in bioapatite structures can lead to the dissolution of endogenous enamel structure and the incorporation of exogenous compounds via ionic exchange processes [25]. Evidence for post‐depositional alteration is further supplied by numerous FTIR indices in the Oldenburg samples falling outside of accepted reference ranges.

Overall, this study demonstrates that while FTIR pre‐screening of enamel samples can reveal certain structural diagenetic alteration, it does not directly indicate modification to the in vivo carbon and oxygen isotope ratios. Furthermore, FTIR analysis has limited capacity to distinguish exogenous carbonate contamination, as soil‐derived carbonates exhibit overlapping absorbance peaks, such as the characteristic 1415 cm^−1^ peak shared by enamel bioapatite and other carbonate minerals (CaCO_3_, MgCO_3_) [83].

## 6 Conclusions

Based on findings from this study as well as previously published data sets, we observe:

- Pre‐treatments meant to remove organic contamination, such as H_2_O_2_ and NaClO, should be avoided as they incur unwanted changes to the chemical composition and isotope values of tooth enamel carbonates.
- Pre‐treatments involving the use of acetic acid, while likely efficient in removing secondary carbonates, impart some changes in enamel carbonate *δ* ^13^C and *δ* ^18^O values. Isotopic shifts observed in both modern and archaeological tooth samples suggest that, in addition to removing exogenous carbonates, acetic acid also dissolves portions of structural carbonate or induces other modifications such as recrystallisation or ionic exchange.
- Both buffered and unbuffered acetic acids are suitable solvents as they affect sample loss, structure and isotope values in a comparable manner.
- While no differences between acetic acid treatment lengths were found in this study, we, in agreement with previous researchers, recommend shorter duration treatments (e.g., 15 min) to minimise the risk of structural enamel carbonate alteration, dissolution, and recrystallisation.

We also conclude that ATR–FTIR pre‐screening, while useful in assessing the structural integrity of the sample and diagenetic alteration, might not directly establish the preservation of in vivo oxygen and carbon isotope ratios. Nonetheless, we strongly recommend ATR–FTIR pre‐screening as a means to document and critically evaluate potential vectors of isotopic change. It is also clear that chemical pre‐treatment–induced changes to *δ* ^13^C and *δ* ^18^O values are influenced by environmental variables and burial sedimentary context, the latter of which may vary across a single archaeological site. Therefore, careful consideration of site formation processes, sediment mineral composition, and perhaps also sediment isotopic composition is essential for understanding the diagenetic pathways affecting archaeological enamel. Finally, we emphasise the urgent need to standardise protocols for the preparation of enamel bioapatite samples to ensure reproducibility of results and minimise interpretative bias.

## Author Contributions

**Karolina Varkulevičiūtė:** conceptualization, methodology, formal analysis, investigation, visualization, writing – original draft, writing – review and editing. **Christine Winter‐Schuh:** conceptualization, methodology, data curation, investigation, writing – review and editing. **Cheryl A. Makarewicz:** conceptualization, methodology, data curation, investigation, supervision, writing – review and editing.

## Peer Review

The peer review history for this article is available at https://www.webofscience.com/api/gateway/wos/peer‐review/10.1002/rcm.10090.

## Supporting information


**Table S1.** Carbon (δ^13^C) and oxygen (δ^18^O) isotope values measured from powdered tooth enamel samples subjected to different pre‐treatment protocols.
**Table S2.** ATR‐FTIR data describing untreated tooth enamel powder samples.
**Table S3.** ATR‐FTIR data describing tooth enamel powder samples subjected to various pre‐treatment protocols.
**Table S4.** Carbon (δ^13^C) and oxygen (δ^18^O) isotope values of concretions and bulk enamel from THS tooth specimens.
**Table S5.** Sample loss statistic

**Figure S1** Example images of teeth condition prior to sampling. (A) THS teeth exhibit visible calcareous concretions up to a few millimetres in thickness; these were removed prior to sampling. (B) Oldenburg LA 77 teeth exhibit visible brown and black discolouration of the enamel and dentin. (C) WF16 teeth display brittle fragmented enamel.
**Figure S2.** FTIR spectra of enamel example (A) and dried 0.1 M acetic acid treatment supernatant (B) from THS (C) Oldenburg LA 77 and (D) WF16.
**Figure S3.** FTIR spectra of calcareous concretions removed from Tell Handaquq South tooth samples. The spectra indicate that the concretions are made up of carbonated apatite.
**Figure S4.** FTIR spectra of sediment from Oldenburg LA 77 (A) and WF16 (B). Oldenburg sediment spectra suggest a high silica content (likely in the form of quartz), gypsum and only very low quantities of carbonates. WF16 spectra suggest a high silica and carbonate content with quartz and gypsum present.

## Data Availability

The data that support the findings of this study are available from the corresponding author upon reasonable request.
