# Supplementary material for: Methodological Considerations for the Use of Acid‐Based Pre‐Treatment Protocols for Carbon and Oxygen Analysis of Tooth Enamel
Source: Rapid Commun Mass Spectrom. 2025 Aug 1;39(20):e10090. doi: 10.1002/rcm.10090 (PMC12314474; doi:10.1002/rcm.10090)

Figure S1. Example images of teeth condition prior to sampling. A. Tell Handaquq South, has visible calcareous concretions that were up to a few mm thick and had to be removed prior to sampling. B. Oldenburg, has visible brown and black discolouration of the enamel and dentin C. Wadi Faynan 16, has thin, brittle fragmented enamel.
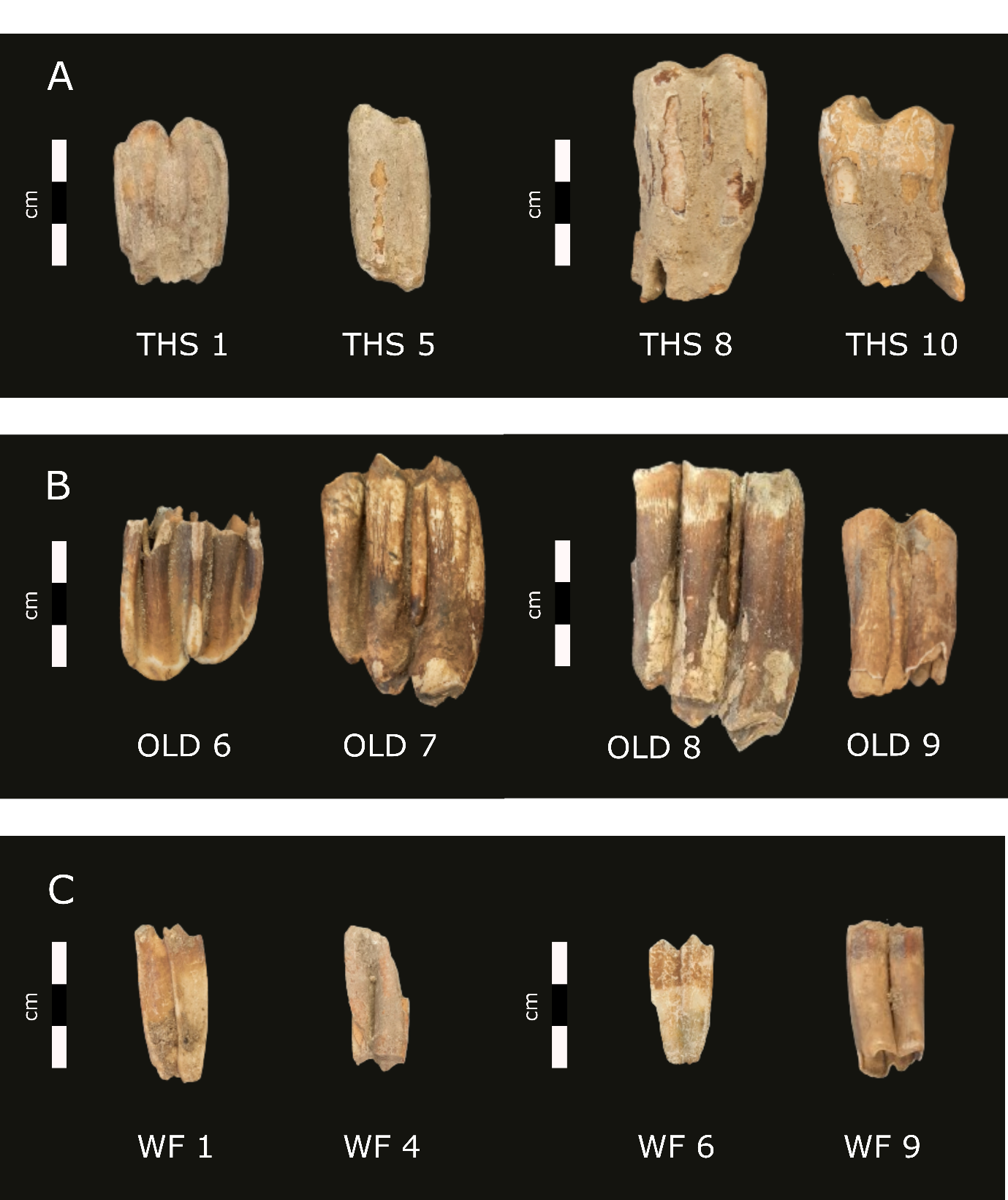


Figure S2. FTIR spectra of enamel example (A) and dried 0.1 M acetic acid treatment supernatant (B-D). B. supernatant from Tell Handaquq South sample, C. supernatant from Oldenburg sample, D. supernatant from Wadi Faynan 16 sample.


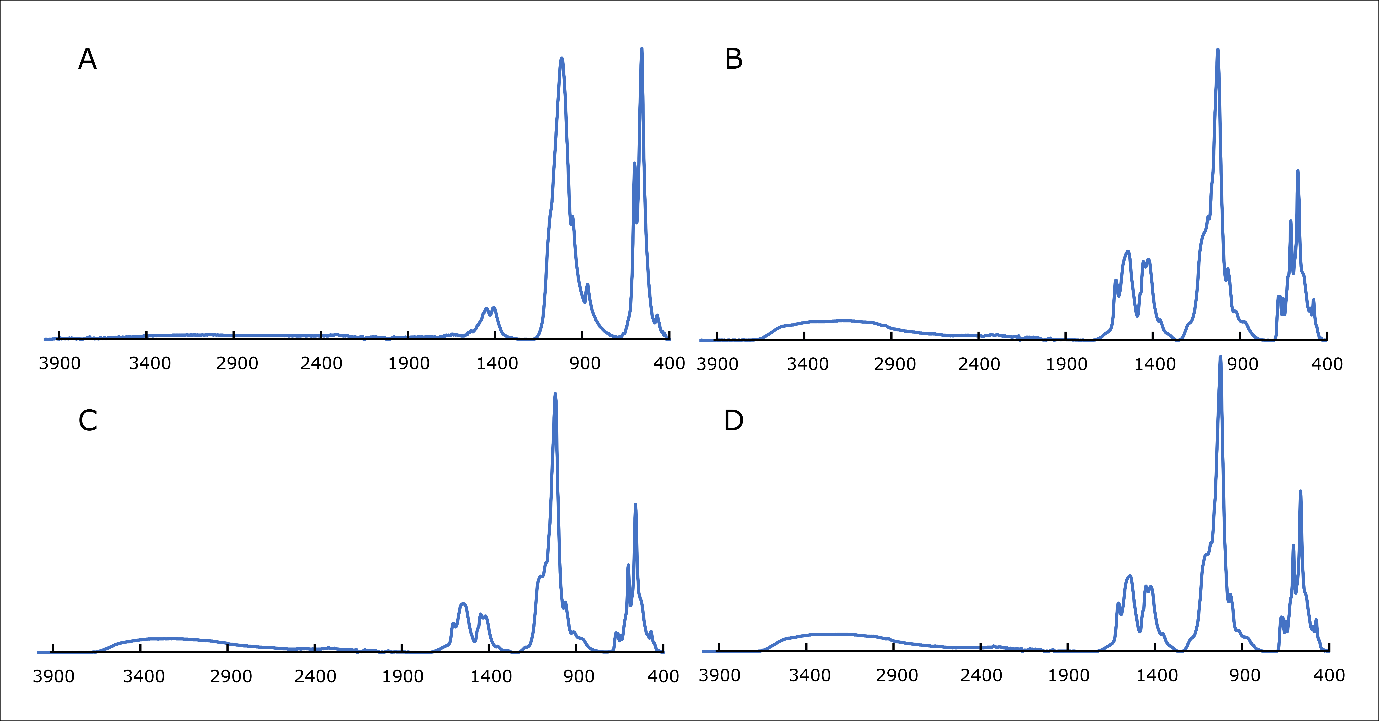


Figure S3. FTIR spectra of calcareous concretions removed from Tell Handaquq South tooth samples. The spectra indicate that the concretions are made up of carbonated apatite.


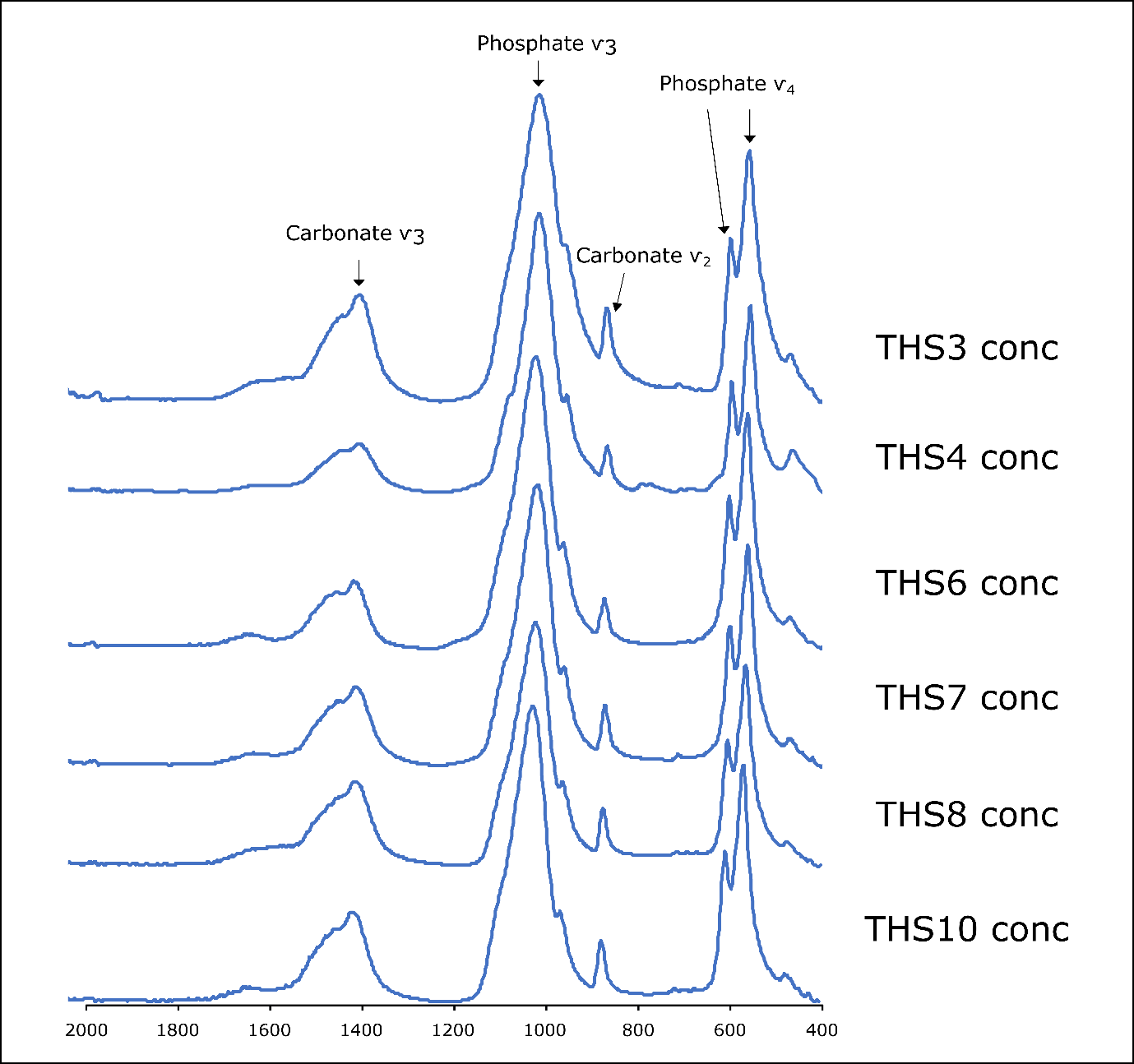


Figure S4. FTIR spectra of sediment from Oldenburg (A) and Wadi Faynan 16 (B). Oldenburg sediment spectra suggests a high silica content (likely in the form of quartz), gypsum, and only very low quantities of carbonates. Wadi Faynan 16 spectra suggests a high silica and carbonate content, with presence of quartz and gypsum.


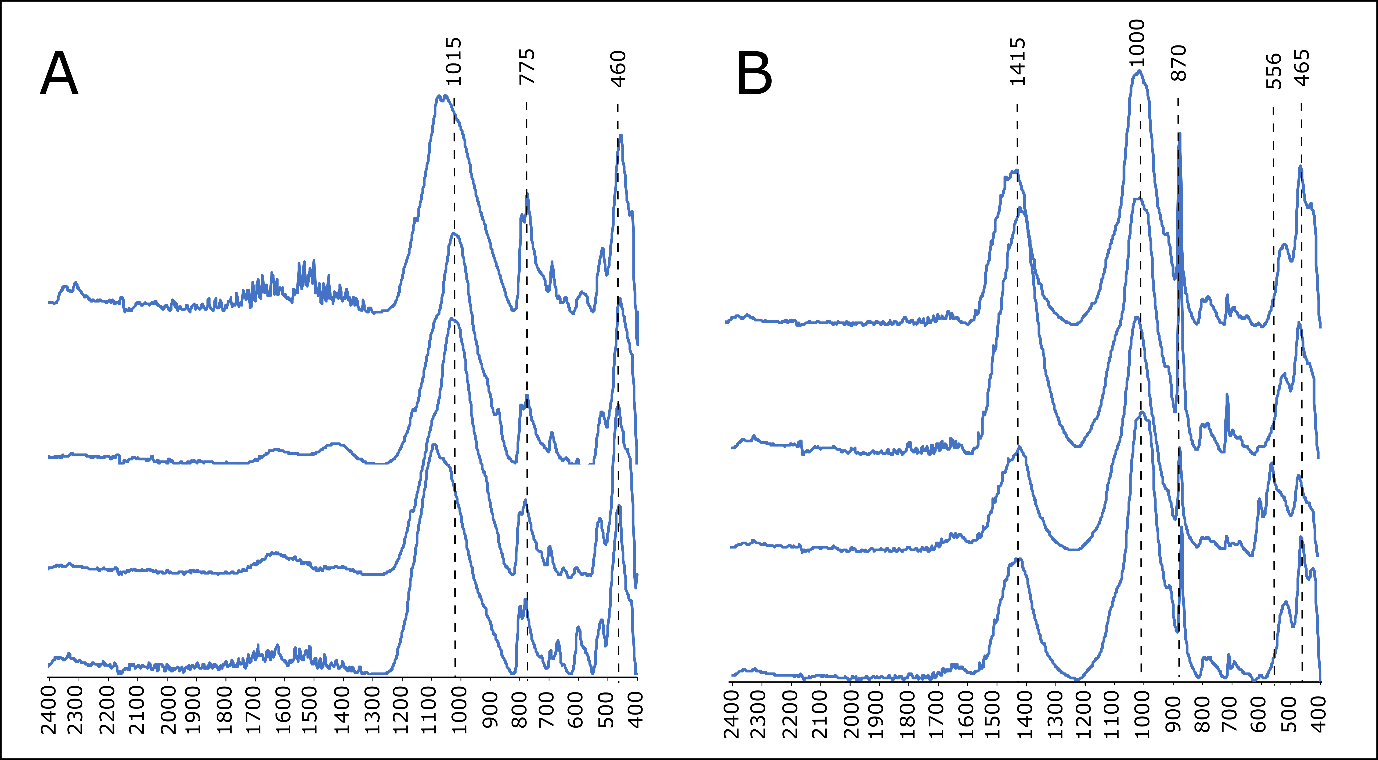

Supplement: Supplementary file 2 — Figure S1 Example images of teeth condition prior to sampling. (A) THS teeth exhibit visible calcareous concretions up to a few millimetres in thickness; these were removed prior to sampling. (B) Oldenburg LA 77 teeth exhibit visible brown and black discolouration of the enamel and dentin. (C) WF16 teeth display brittle fragmented enamel. Figure S2. FTIR spectra of enamel example (A) and dried 0.1 M acetic acid treatment supernatant (B) from THS (C) Oldenburg LA 77 and (D) WF16. Figure S3. FTIR spectra of calcareous concretions removed from Tell Handaquq South tooth samples. The spectra indicate that the concretions are made up of carbonated apatite. Figure S4. FTIR spectra of sediment from Oldenburg LA 77 (A) and WF16 (B). Oldenburg sediment spectra suggest a high silica content (likely in the form of quartz), gypsum and only very low quantities of carbonates. WF16 spectra suggest a high silica and carbonate content with quartz and gypsum present. [file RCM-39-e10090-s001.docx]
